# Chikungunya virus infection in Italy: epidemiology, climate change implications and public health recommendations

**DOI:** 10.3389/fpubh.2026.1791544

**Published:** 2026-05-08

**Authors:** Pasquale Stefanizzi, Pierluigi Lopalco, Viviana Balena, Viviana Vitale, Giuseppina Iannelli, Domenico Martinelli, Stefano Termite, Francesca Centrone, Maria Chironna, Francesca Fortunato

**Affiliations:** 1 Interdisciplinary Department of Medicine, University of Bari Aldo Moro, Bari, Italy; 2 Department of Experimental Medicine, University of Salento, Lecce, Italy; 3 Health Prevention Department, Local Health Authority of Foggia, Foggia, Italy; 4 Department for the Promotion of Animal Health and Welfare - Section for the Promotion of Health and Safety in the Workplace, Bari, Italy; 5 Department of Medical and Surgical Sciences, University of Foggia, Foggia, Italy; 6 Health Prevention Department, Local Health Authority of Brindisi, Brindisi, Italy; 7 Laboratory of Public Health, AOUC Policlinico, Bari, Italy; 8 Interdisciplinary Department of Medicine, Laboratory of Public Health AOUC Policlinico, Bari, Italy

**Keywords:** control strategies, risk communication, travel medicine, vaccination, vector infectious disease

## Abstract

Chikungunya is an emerging public health threat in Europe, driven by climate change, vector expansion and international travel. Italy has recently experienced an increasing proportion of autochthonous cases, highlighting gaps in surveillance, prevention and preparedness. Recent national and regional data, including those from Apulia Region, confirm ongoing transmission risk in receptive areas. Integrated surveillance, vector control, risk communication and targeted vaccination strategies are available, although unevenly implemented. This policy brief evaluates current evidence and outlines actionable recommendations to support timely decision-making and reduce the risk of local Chikungunya outbreaks.

## Background

Chikungunya virus (CHIKV) is a *Togaviridae* family arbovirus transmitted by mosquitoes of the *Aedes* genus (1). CHIKV infection’s manifestation consists in an acute febrile disease known as Chikungunya fever, sometimes coming with a rash, myalgia and severe multi-joint arthralgia. Although usually self-limiting, the joint pain associated with the disease may become chronic and lead to invalidating forms of rheumatic disease. This outcome significantly impacts the patient’s quality of life (2).

Although considered a neglected disease confined to endemic areas in Africa (e.g., Tanzania, South Africa, Zimbawe, Democratic Republic of Congo) and Asia (e.g., Thailand, Southeast Asia, India), Chikungunya fever has been causing growing concern since 2004, when outbreaks were identified outside traditionally endemic areas (e.g., Kenya, Indian Ocean and American islands, Central and West Africa, and Europe) (3). Until recently, cases identified in high-income countries have been exclusively imported from endemic territories via international travel; however, an increase in autochthonous infections is currently being observed in high-income countries themselves. The growing worry that CHIKV might widen its international spread has concentrated research endeavours towards developing new immunisation products (4).

The first CHIKV vaccine has been approved by the United States Food and Drug Administration (FDA) in 2023. It is a live attenuated product, to be administered in a single dose. The same vaccine has been authorised for marketing by the European Medicines Agency (EMA) in June 2024 (5). To date, the vaccine has not been approved by the Italian Drug Agency (AIFA).

In February 2025, both FDA and EMA authorised a second vaccine against CHIKV (6). The vaccine has been approved by AIFA in May 2025, and has been made available since October 30th, 2025 (7). It is a recombinant adsorbed vaccine based on virus-like particle (VLP) technology, dedicated to subjects aged twelve or older and administered in single dose. Efficacy estimates have been based on neutralising CHIKV antibody titres, reaching 84.0% after 183 days in subjects between 12 and 64 years (8) and 74.4% in subjects older than 64 (9). Both clinical studies (8, 9) and field evaluations (10) have shown a favourable safety profile for this vaccine.

In its December 23th, 2025, update, the Italian National Institute of Health (ISS) reported 469 confirmed Chikungunya infection cases in 2025. Out of them, 384 were locally-acquired, while 85 were linked to international travel. No deaths were registered. Local CHIKV transmission was documented in four Italian Regions (Emilia-Romagna, Veneto and Toscana) (11).

On December 17st, 2025, the European Centre for Disease Prevention and Control (ECDC) highlighted that only two European countries, Italy and France, have reported cases of Chikungunya fever during 2025. For comparison, France reported 788 cases throughout the year. All clusters1 reported in France are now closed. In Italy, only one cluster remains open (in Bellaria-Igea Marina) (12).

From September to December 23rd, 2025, six cases of CHIKV infection were confirmed in Apulia, a region in south-eastern Italy. Five of the patients lived in Bari, the region’s main province, while the other lived in the Barletta-Andria-Trani province. Epidemiological investigations revealed that five of the cases were linked to travel to Cuba, while one was imported from Sri Lanka. Two patients required hospitalisation. Another case was classified as possible. This individual had travelled to a neighbouring region (Basilicata) during the likely exposure period. However, CHIKV circulation was not confirmed in Apulia. No confirmed cases were reported in Apulia in the years 2021–2024 according to national surveillance data. This low incidence should not be interpreted as absence of risk, but rather as an indicator of a receptive setting where sporadic importations may occur without necessarily leading to sustained transmission. In Italy, previous outbreaks have demonstrated that even a limited number of imported cases may trigger local transmission in the presence of competent vectors and favourable environmental conditions (13–17).

The increase of the local-to-imported cases’ proportion is concerning. Although the reasons for this phenomenon have not been fully investigated yet, it could be related to various changes that have been occurring over the last decade. First of all, the ongoing Western Mediterranean basin’s subtropicalization is likely to improve the environmental fitness of *Aedes* mosquitoes, leading to a wider vector circulation (18).

Secondly, certain species belonging to the *Aedes* genus are known for their ecophysiological plasticity. Indeed, these arthropods can enter a physiological state of suspended development and reduced metabolic activity – a phenomenon known as “overwintering” – which grants them better resistance to winter temperatures. This adaptation, increasingly observed in certain species of *Aedes* mosquitoes, allows them to spread to colder ecoregions, a trend driven by climate change (19).

Finally, the post-pandemic years have been characterised by an increase of touristic outflows to exotic destinations compared to pandemic years—and, for certain destinations, exceeding the pre-pandemic baseline—alongside a surge in migratory inflows towards Europe that significantly exceeds pre-pandemic levels (20, 21). CHIKV’s spread might have been facilitated by these population movements, leading to infection of locally well-established vector populations (22).

The United States Centers for Disease Control and Prevention (CDC) currently recommend vaccination with the recombinant adsorbed product for adolescents and adults aged twelve or older in case of travel to zones currently involved in a Chikungunya outbreak, as well as countries or territories deemed as high-risk for travellers. In the latter case, only stays lasting over 6 months are considered at risk for CHIKV infection. The vaccine is also recommended to laboratory technicians who might be exposed to the virus (23).

In Germany, the Permanent Commission on Vaccines and the German Tropical Medicine, Travel Medicine and Global Health Society recommend vaccination to subjects over twelve travelling to an area where a Chikungunya outbreak is currently taking place, as well as travellers planning to either stay for more than 4 weeks or take short-term, but repeated trips to endemic areas. Vaccination is especially recommended to subjects at an increased risk of severe Chikungunya fever and/or chronic pain, such as people over 60 and comorbid patients, as well as workers at an occupational risk of CHIKV exposure, such as research centres and laboratory staff (24).

The United Kingdom Joint Committee on Vaccination and Immunisation (JCVI) shares the Chikungunya vaccine recommendation as far as travellers to areas with active CHIKV outbreaks. It also recommends the vaccine in case of long-term or frequent travels to areas where CHIKV transmission was documented at least once over the last five years and to laboratory staff who may be exposed to the virus (25). In Italy, specific recommendations on chikungunya vaccination have so far been issued mainly by the Italian Society of Travel and Migration Medicine (SIMVIM), which supports the use of the vaccine in travellers to areas with active outbreaks or documented transmission in recent years, particularly in subjects at increased risk of severe disease and in occupationally exposed personnel, with the aim of ensuring individual protection and reducing the risk of imported cases that may lead to local transmission in receptive areas (26).

Chikungunya represents not only a clinical issue but also a priority for public health policy, requiring coordinated strategies for surveillance, prevention and response. This policy brief focuses on Italy, with particular reference to the Apulia Region, to evaluate current challenges and propose evidence-based policy options that support preparedness and public health planning in a receptive region where the current burden is limited, but where environmental and epidemiological conditions for local transmission are present.

## Policy options and implications

Policy option 1: Strengthening integrated surveillance. Combining epidemiological, laboratory and entomological data in integrated surveillance is a cornerstone of early outbreak detection. National data from 2025 show that a substantial proportion of cases are locally acquired, highlighting the importance of timely notification and standardised case definitions.

Implications: Strengthening integrated surveillance requires moderate resources but offers a high impact by enabling a rapid response and reducing the size of outbreaks and the associated healthcare costs. Experience from integrated arbovirus surveillance programs in Italy, particularly those developed for West Nile Virus within a One Health framework, has shown that combining human, animal, and vector surveillance allows earlier detection of viral circulation, supports targeted interventions, and reduces overall public health costs, including those related to blood and organ donor screening. Such evidence highlights the potential value of integrated surveillance systems for emerging arboviruses like chikungunya (27).

Policy option 2: Enhancing vector control strategies. Structural interventions to hinder the vectors’ spread or survival, especially in case of local outbreaks, include environmental management, disinfestation of the case’s surroundings via bug-killing agents, favouring substances that kill adult mosquitoes both in public land and private properties, and door-to-door inspections to locate and eliminate larval reservoirs in the vicinities of houses. As a future perspective, strategies demonstrated to be effective for Dengue fever outbreak containment (e.g., the introduction of genetically modified male mosquitoes inducing sterility in wild females and the infection of larvae with Wolbachia genus bacteria (28, 29)) could be considered, albeit consolidated evidence of effectiveness against Chikungunya outbreaks is not currently available. In addition to vector control strategies, international health authorities still underline the importance of behavioural strategies to prevent mosquito bites, which are recommended to international travellers heading to endemic areas, subjects living in close vicinity to Chikungunya cases, and CHIKV-infected subjects during the acute phase of the disease, in order to avoid infection of receptive mosquitoes. These include wearing protective clothing; ensuring repeated application of mosquito repellents on exposed body parts and clothes; sleeping in rooms screened by mosquito nets and/or equipped with air conditioning, avoiding sleeping in the open air; preventing water stagnation nearby households or, when impossible, treating stagnation points with mosquito repellents; making sure that all reservoirs of drinkable water nearby households are fully covered at any time (30–33). Implications: Although vector control is effective, its long-term effectiveness depends on intersectoral coordination, engagement from local authorities and public compliance. A reactive-only approach may limit long-term effectiveness.

Policy option 3: Risk communication and community engagement. Targeting risk communication at residents, travellers and contacts of confirmed cases can significantly reduce exposure to mosquito bites and prevent secondary transmission (34, 35). Implications: Although communication strategies are low-cost and highly acceptable, they require consistent messaging and integration into routine public health activities.

Policy option 4: Strategic vaccination to prevent virus importation and protect vulnerable populations. The recent authorisation of CHIKV vaccines by international regulatory agencies provides a new preventive tool, particularly for travellers heading to endemic areas, who may introduce the virus in non-endemic countries, leading to autochronous transmission. Furthermore, vaccines represent a possible strategy to protect local high-risk groups, for whom targeted vaccination programs may be implemented in the event of an outbreak with demonstrated local transmission. Implications: Clear national guidance is needed to ensure equitable access and appropriate targeting. This requires integration with existing travel medicine services, as well as established protocols for offering the vaccine during local public health emergencies.

Policy option 5: Integrating climate change adaptation and predictive modelling. Rising temperatures and altered precipitation patterns are promoting the geographical and seasonal spread of *Aedes* mosquitoes. The development of early warning systems that cross-analyze meteorological data with epidemiological and entomological surveillance would be highly valuable in this context. Additionally, urban planning would be crucial to minimise new breeding sites triggered by extreme weather events. Implications: Building predictive climate-health models requires multidisciplinary collaboration and significant resource allocation. However, in the long run, this approach would enable the rapid implementation of vector control strategies when climate anomalies indicate an elevated risk.

## Recommendations

Considering the current epidemiological scenario and the strategies defined by healthcare authorities worldwide, the following is recommended:

Priority 1 - surveillance & early detection

- Ensure timely and full implementation of regional, national and international flow of information by rapidly notifying Chikungunya cases to competent surveillance systems.
- Strengthen integrated epidemiological and entomological surveillance systems by sharing data in a timely manner at regional and national levels.
- Ensuring timely diagnosis by employing common definitions: laboratory-based diagnosis of Chikungunya fever must be taken into consideration for all cases presenting acute onset of fever >38.5 °C and severe or invalidating arthralgia unexplained by other medical conditions, in subjects living in areas with documented local transmission or who recently visited endemic areas; recommended protocols include reverse transcriptase-polymerase chain reaction (RT-PCR) on serum, up to the fifth day following onset of symptoms, as well as serological IgM/IgG assays (starting six days after the onset).
- Training healthcare personnel on differential diagnosis among Chikungunya, Dengue and Zika virus infection, given the similar symptoms and the risk to misdiagnose subjects. Healthcare workers should be sensitised about the importance of laboratory diagnosis and epidemiological investigations.
- Defining protocols describing clinical follow-up, contact tracing and surveillance of vectors.

Priority 2 – vector control & environmental management

- In case of further epidemiologically linked cases, considering disinfestation of the outbreak-affected area via airborne insecticides, following existing guidelines and safety protocols for chemical agent dispersal.
- Installing and/or refurbishing mosquito nets in hospitals, in order to safeguard hospitalised patients with specific risk factors for severe CHIKV infection and complications.
- Strengthening local planning on the removal of mosquito proliferation sites, involving both the public and political stakeholders.
- Strengthening entomological surveillance by placing sentinel traps and monitoring the presence and density of mosquitoes of the Aedes genus.
- Promote collaboration between public health authorities, municipalities, and entomologists.
- Involving biologists and entomologists in epidemiological investigations related to Chikungunya fever suspects, and whenever the environment’s characteristics are deemed favourable to the spread of mosquitoes. In this context, these professionals can contribute by conducting environmental assessments of animal habitats to identify mosquitoes breeding sites and utilising individual animals as sentinels for vector activity.

Priority 3 – risk communication & travel medicine

- Reinforcing travel medicine counselling, systematically including information and risk communication about CHIKV in routine counselling.
- Providing recommendations not only to travellers, but also to contacts of locally-acquired cases; contacts should be considered at an especially high risk when sharing living quarters and/or water reservoirs with the cases (water reservoirs must be deemed at risk whenever they allow for the development of *Aedes* mosquitoes’ larvae).
- Enact prevention protocols to avoid that infected subjects come into contact with receptive arthropodal vectors, especially during the acute febrile phase of disease, based on systematic deployment of mosquito repellents both indoors and outdoors, use of full-body clothing and mosquito net placement/maintenance.
- Reinforcing public communication via targeted mass media campaigns, providing clear information about how to prevent mosquito bites and how to handle the vector’s reproduction sites.

Priority 4 – vaccination strategies

- Ensuring that vaccination is promptly available to subjects travelling to endemic areas and territories currently involved in Chikungunya outbreaks.

## Conclusion

Chikungunya is a preventable yet growing public health threat in Italy and other European countries. Even in regions where only sporadic cases are currently reported, the presence of competent vectors and increasing international mobility justify preparedness measures. The effectiveness of outbreak control may be limited by fragmented surveillance and reactive interventions. Evidence-based, coordinated policies that integrate surveillance, vector control, vaccination and risk communication are essential in reducing the likelihood and impact of local transmission. This policy brief provides practical options and actionable recommendations to support informed decision-making and strengthen public health preparedness.
